# Computational analysis of R–X oxidative addition to Pd nanoparticles†

**DOI:** 10.1039/d4sc00628c

**Published:** 2024-05-24

**Authors:** Mikhail V. Polynski, Yulia S. Vlasova, Yaroslav V. Solovev, Sergey M. Kozlov, Valentine P. Ananikov

**Affiliations:** a Department of Chemical and Biomolecular Engineering, National University of Singapore 4 Engineering Drive 4 Singapore 117585 Singapore polynskimikhail@gmail.com mvp@nus.edu.sg; b Faculty of Chemistry, Moscow State University Leninskiye Gory 1-3 Moscow 119991 Russia; c Zelinsky Institute of Organic Chemistry, Russian Academy of Sciences Leninsky Prospect 47 Moscow 119991 Russia val@ioc.ac.ru; d M. M. Shemyakin and Yu. A. Ovchinnikov Institute of Bioorganic Chemistry of the Russian Academy of Sciences Miklukho-Maklaya 16/10 Moscow 117997 Russia

## Abstract

Oxidative addition (OA) is a necessary step in mechanisms of widely used synthetic methodologies such as the Heck reaction, cross-coupling reactions, and the Buchwald–Hartwig amination. This study pioneers the exploration of OA of aryl halide to palladium nanoparticles (NPs), a process previously unaddressed in contrast to the activity of well-studied Pd(0) complexes. Employing DFT modeling and semi-empirical metadynamics simulations, the oxidative addition of phenyl bromide to Pd nanoparticles was investigated in detail. Energy profiles of oxidative addition to Pd NPs were analyzed and compared to those involving Pd(0) complexes forming under both ligand-stabilized (phosphines) and ligandless (amine base) conditions. Metadynamics simulations highlighted the edges of the (1 1 1) facets of Pd NPs as the key element of oxidative addition activity. We demonstrate that OA to Pd NPs is not only kinetically facile at ambient temperatures but also thermodynamically favorable. This finding accentuates the necessity of incorporating OA to Pd NPs in future investigations, thus providing a more realistic view of the involved catalytic mechanisms. These results enhance the understanding of aryl halide (cross-)coupling reactions, reinforcing the concept of a catalytic “cocktail”. This concept posits dynamic interconversions between diverse active and inactive centers, collectively affecting the outcome of the reaction. High activity of Pd NPs in direct C–X activation paves the way for novel approaches in catalysis, potentially enhancing the field and offering new catalytic pathways to consider.

## Abstract 2

A computational elucidation of the mechanism of R–X oxidative addition to Pd nanoparticles revealed high activity at the edges of nanoparticles and identified key intermediates before and after oxidative addition.

## Graphical abstract

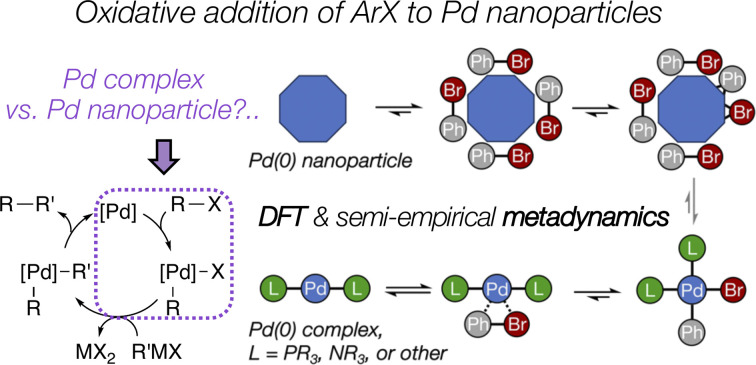

## 1. Introduction

Facile synthesis of functionalized organic molecules is of paramount importance for the development of personalized medicine, fine chemical synthesis, advancements in drug design, and innovative materials, among many other areas. Within this conceptual demand, activation of the carbon–halogen (C–X) bond in organic halides (R–X) with subsequent transfer and functionalization of the organic group (R) is the basis for many well-recognized synthetic approaches known for their cost-efficiency, wide scope, and general applicability.^1–3^ Activation of the C–X bond occurs through oxidative addition (OA), initiating a series of transformations in catalyst active centers.

Developing synthetic methodologies involving OA as an essential step in the reaction mechanism uncovered dynamic catalyst interconversions as a common phenomenon.^4^ Dynamic catalyst interconversions are now at the forefront of catalysis as the key concept to developing a new generation of synthetic technologies and addressing sustainability problems.^4–7^ This dynamic behavior was exceptionally evident in one of the cornerstone classes of organic reactions, Pd-catalyzed (cross-)couplings.^8,9^ Previous studies have shown that a “cocktail” of dynamically interconverting Pd species can easily form under catalytic conditions, including metal complexes, halides, and nanoparticles as catalytically active centers or pre-activated Pd reservoirs, with their activity varying tremendously in some cases.^10–17^ The dynamic phenomena occur under homogeneous and heterogeneous catalysis conditions.^7^ It has been demonstrated that oxidative addition of aryl halides plays a central role in the formation of “cocktail”-type systems.^17,18^

OA to Pd(0) metal complexes was investigated in experimental, DFT, and combined experimental/theoretical studies.^19–28^ However, the high level of reaction mechanism complexity still drastically challenges our understanding of OA, even at the level of molecular complexes.^29^

Pd NPs have shown remarkable activity in cross-coupling reactions as versatile and practical catalysts. In many studies, the activity was associated with Pd leached into the solution, not with the surface reactions on Pd NPs.^9,30–34^ At the same time, Pd nanoparticles are often formed spontaneously during cross-coupling reactions.^35–39^

While OA plays a key role in Pd leaching,^17^ its mechanism on the nanoparticle surface remains unexplored since previous computational and experimental–computational studies focused on energies of Pd detachment from the NP surface or cross-coupling reactions on molecular subnanoclusters.^40–45^ In the present study, for the first time, we investigated the oxidative addition of an organic halide to the Pd surface at the nano-scale. The studied process is relevant to C–C and C–heteroatom bond formation in applied fine organic synthesis involving nanocatalysts. Comparison with regular Pd complexes, including various ligands, highlighted a critical difference in C–X bond activation at the molecular and nano-scale levels.

## 2. Results

**Fig. 1:**
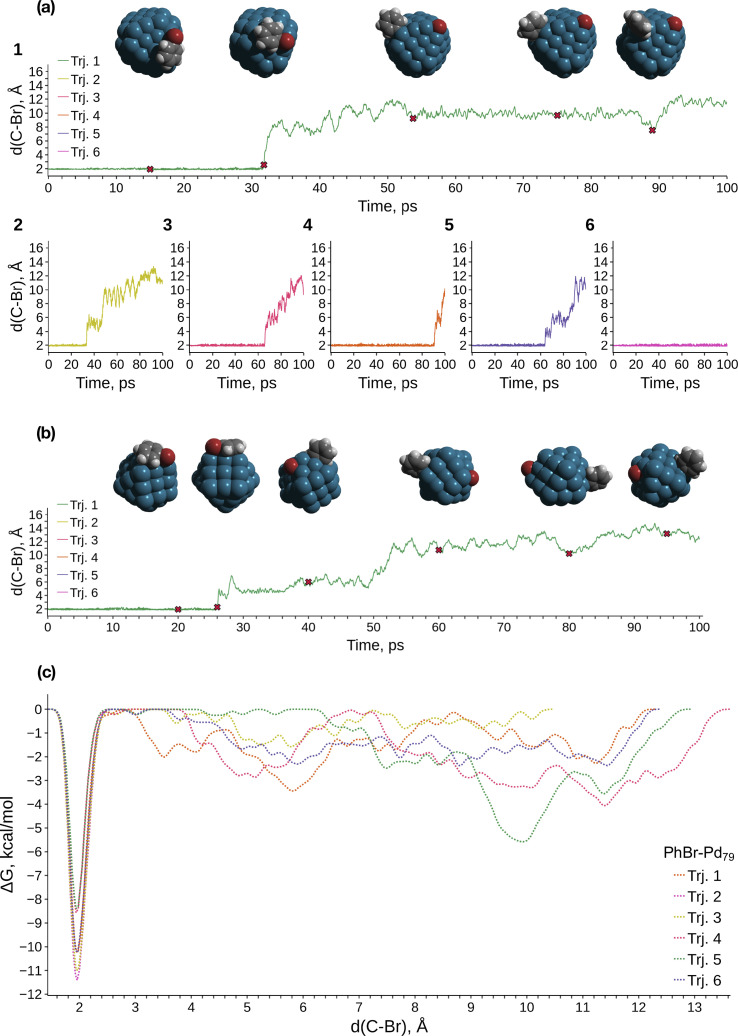
(a) Collective variable (C–Br distance) evolution during the metadynamics simulations of the Pd_79_ system; (b) selected case of the collective variable evolution in the Pd_55_ system; (c) free energy profiles obtained from the metadynamics runs of PhBr OA to Pd_79_. The color scheme is as follows: Pd – dark cerulean; Br – dark red; C – grey; H – white.

We conducted metadynamics (MTD) modeling of oxidative addition (OA) to truncated octahedral Pd_79_ and Pd_140_ nanocrystallites, along with a smaller Pd_55_ nanocrystallite, using the GFN1-xTB Hamiltonian (Pd_79_ and Pd_55_: Fig. 1; Pd_140_: Fig. S2†). We observed OAs in almost all Pd_55_ and Pd_79_ systems within tens of picoseconds (Fig. S1a† and 1a, respectively). A representative case of the PhBr OA to Pd_79_ is depicted in Fig. 1a(1). PhBr dissociation occurred at the edge of the nanoparticle. Subsequently, the Br atom migrated to the (1 0 0) facet and remained bonded to it. Concurrently, the Ph group exhibited active migration along the edge between two (1 1 1) facets and the adjacent (1 0 0) facet, as illustrated in the snapshot structures at the top of Fig. 1a.

The OA mechanism and post-OA system evolution were similar across all cases examined. The majority of OA events occurred at nanoparticle edges. In a few cases involving Pd_55_, the OA occurred at the vertex of the nanoparticle, which is also a site comprised of a Pd atom with a low coordination number (see details below). Only one OA to Pd_140_ was observed within 100 ps of sampling due to the increased configuration space size; this reaction also occurred on the edge (Fig. S2†). In the Pd_55_ and Pd_140_ systems, Ph migrated along the NP edges similarly to the case of Pd_79_ throughout all MTD simulations (Fig. 1a, b and S2a†). After the OA, Br similarly remained tightly bound to one of the (1 0 0) facets of Pd_140_. In contrast, some mobility of the adsorbed Br was observed on Pd_55_, with Br migrating along the (1 0 0) facet, which is larger in Pd_55_ than in Pd_79_ and Pd_140_ (Fig. 1b).

Fig. 1c presents the free energy profiles (FEPs) obtained from individual MTD runs involving Pd_79_. The movement of the dissociated Ph along the edge corresponds to a series of shallow minima on the right side of the obtained FEPs. Although these basins are deeper than the “thermal energy”, RT, at 298.15 K (0.6 kcal mol^−1^), Ph migration occurs relatively easily. The corresponding FEP minima are significantly higher than the lowest sharp minimum at approximately 1.9 Å, which corresponds to the undissociated state of PhBr. According to the obtained FEP, the OA activation free energy does not exceed approximately 11 kcal mol^−1^, and OA is kinetically feasible at ambient temperature. OA can lead to the accumulation of tightly bound Br atoms on the Pd surface, with a preference for the (1 0 0) surface. This is accompanied by the chemisorption of Ph groups, which migrate easily near low-coordinate Pd atom sites such as edges, steps, vertices, *etc.*

**Fig. 2:**
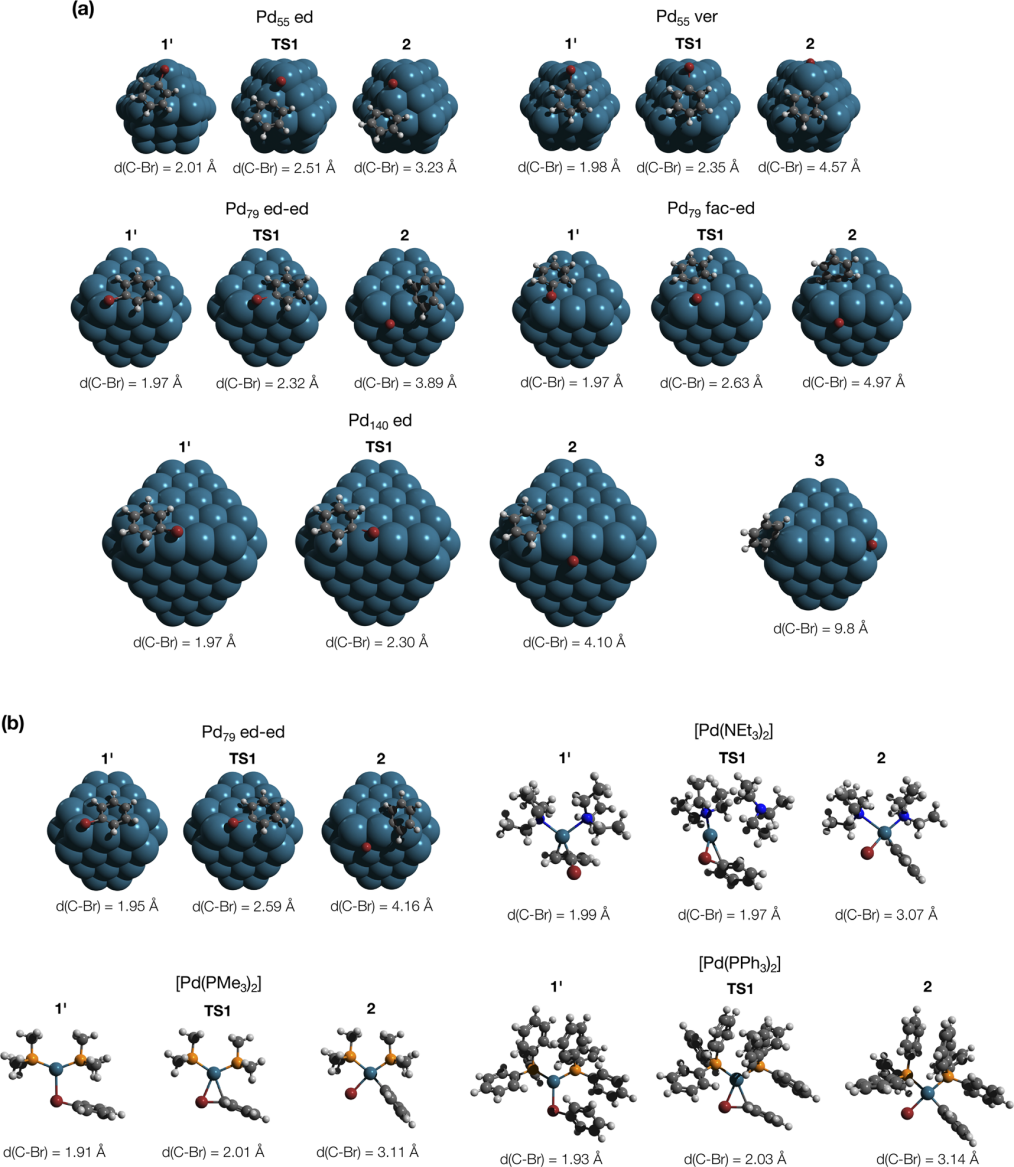
(a) Optimized structures of the OA transition states along with pre-reaction and post-reaction states in the PhBr interactions with Pd_55_, Pd_79_, and Pd_140_ nanoparticles, derived from metadynamics simulations. Intermediate 3, identified during the MTD sampling, is discussed in the text. (b) Optimized structures of the intermediates in the pathways involving Pd_79_ (facet active site) and [PdL_2_] (L = NEt_3_, PMe_3_, and PPh_3_), elucidated in nudged elastic band calculations. The color scheme is as follows: Pd – dark cerulean; Br – dark red; P – orange; N – blue; C – grey; H – white. Intermediate and transition states are numbered according to the scheme in Fig. 3. All optimizations were performed using DFT (see Section S1† for details).

MTD sampling of dissociation trajectories allowed us to identify transition states relevant to modeled nanoparticles. Upon examining the MTD trajectories, we determined two groups of OA processes in the Pd_79_ system occurring at the edge of (1 1 1) facets, with variations in the positioning of the Ph group (Fig. 2a). In the first group, the Ph group was situated on the (1 1 1) facet with the Ph–Br bond nearly perpendicular to the edge (Pd_79_ fac–ed), whereas in the second group, the Ph moiety was positioned such that the Ph–Br bond nearly collinearized with the edge (Pd_79_ ed–ed). The OA of PhBr to Pd_55_ proceeded *via* two distinct pathways: in the first, OA occurred on the edge (labeled Pd_55_ ed in Fig. 2a), and in the second, the vertex Pd atom acted as the reactive center (labeled Pd_55_ ver). The TS found during the MTD sampling of the PhBr–Pd_140_ interaction (Pd_140_ ed in Fig. 2a) was fully similar to that in the Pd_79_ system.

In addition to the OA pathways identified in MTD simulations, we employed the DyNEB method^46^ to explore several alternative active centers. Firstly, we examined OA to Pd–phosphine complexes: the electron-donating PMe_3_ and the widely used relatively weakly electron-donating PPh_3_. Secondly, we included [Pd(NEt_3_)_2_] into the consideration to model a nitrogen base added in some coupling reactions conducted under “ligandless” conditions (the Heck reaction and others). Lastly, using the DyNEB method, we investigated a hypothetical pathway in which the reaction occurred on a facet of the Pd_79_ nanoparticle (labeled Pd_55_ ed in Fig. 2b). Although the latter pathway was not observed in the MTD simulations, it can be used a valuable reference to enhance our understanding of the effects of nanostructuring on OA to Pd catalysts.

**Fig. 3:**
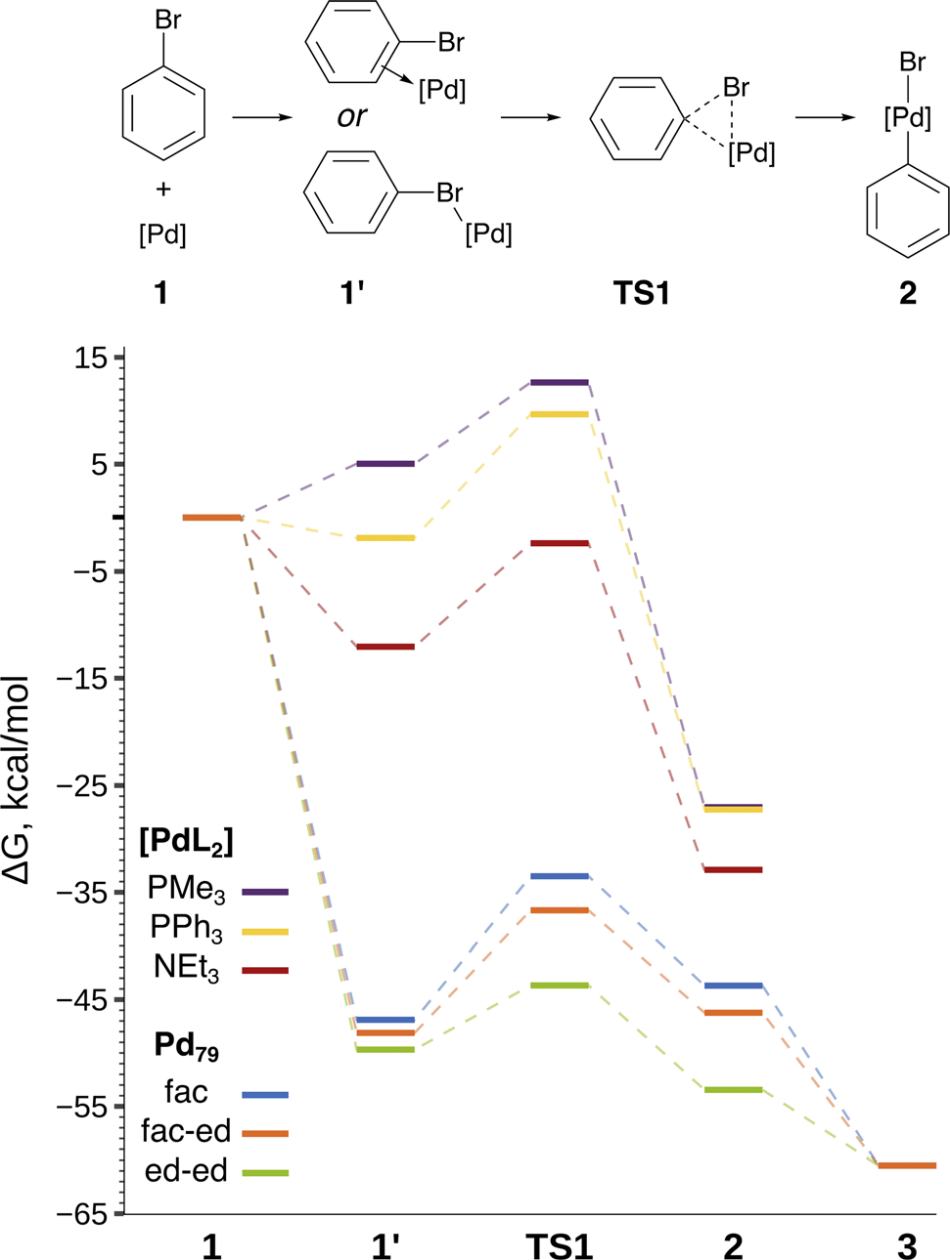
Reaction scheme and the free energy profile. Optimized structures of the intermediates are shown in Fig. 2. See Table S1† for numerical data.

The focal point of this study is the computed free energy profiles of OA to Pd_79_ and molecular complexes depicted in Fig. 3. The corresponding numerical values are given in Table S1.† The process begins with the formation of the pre-OA complex 1′. Pd_79_ was selected to compare the adsorption affinity of PhBr towards Pd nanoparticles *vs.* the thermodynamic effect of 1 → 1′ in the case of molecular complexes [PdL_2_]. The adsorption of PhBr onto Pd_79_ is markedly exergonic, with Δ*G*_1→__1′_ being lower than −46.9 kcal mol^−1^. This result indicates a probable intensive coverage of Pd NP precatalysts by the aryl halide during coupling reactions.

The transition 1 → 1′ is endergonic for [Pd(PMe_3_)_2_] (Δ*G*_1→__1′_ = 5.0 kcal mol^−1^) and exergonic for [Pd(NEt_3_)_2_] and [Pd(PPh_3_)_2_], with Δ*G*_1→__1′_ being equal to −12.0 and −1.9 kcal mol^−1^. In the case of phosphine ligands, PhBr in 1′ binds to [PdL_2_] only through the Br atom, and there is considerable translational and rotational entropy loss. For 1′ with L = NEt_3_, a strong Pd–π bond forms between the aryl halide and the [PdL_2_] moiety, effectively counterbalancing the entropic loss.

The comparison of the DFT-calculated activation energies of oxidative addition is given in Table S1,† and the corresponding plot is given in Fig. 3. Since the transition 1 → 1′ was exergonic in the cases of Pd_79_, [Pd(NEt_3_)_2_], and [Pd(PPh_3_)_2_], we regarded 
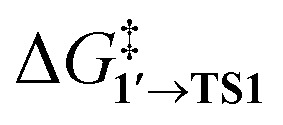
 as the OA activation energy for these species. For [Pd(PMe_3_)_2_], however, 
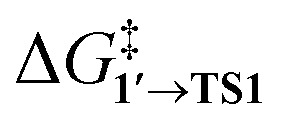
 was considered as the activation energy instead of 
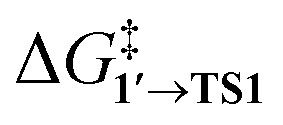
 due to the positive value of Δ*G*_1→__1′_.

In all the molecular complexes, classic three-center Br–Pd–C transition states were identified (Fig. 2), characterized by imaginary modes representing the migration of Ph to Pd and the Ph–Br bond cleavage. The activation barriers of the OA to [Pd(PMe_3_)_2_] and [Pd(PPh_3_)_2_] did not surpass 12.6 kcal mol^−1^, aligning well with the typically facile OA to complexes of Pd(0) with two non-overly sterically hindered phosphine ligands. The OA of PhBr to [Pd(PPh_3_)_2_] proceeded with 
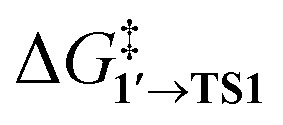
 equal to 9.7 kcal mol^−1^. It is important to note the detachment of the second NEt_3_ from the Pd center in the transition state, whereas both the initial and final states, 1′ and 2, featured NEt_3_ attached to Pd.

The calculated activation energies of PhBr OA to Pd_79_ are comparable to or lower than those involving molecular Pd complexes, with 
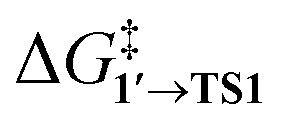
 being equal to 13.4, 11.4, and 6.0 kcal mol^−1^ for Pd_79_ fac, Pd_79_ fac–ed, and Pd_79_ ed–ed, respectively. Evidently, the edge is a highly reactive site with the activation barrier for the Pd_79_ ed–ed transition state well below those observed with the [PdL_2_] complexes. Furthermore, the formation of the pre-reaction state 1′ in pathway Pd_79_ ed–ed is also the most exergonic among these three model reaction channels. Therefore, we may expect the preferable reactivity *via* the ed–ed channel. It should also be noted that the (1 1 1) facet exhibited the lowest activity in the model OA process, indicating that oxidative addition preferentially occurs at the edges of Pd nanoparticles.

Fig. 3 and the last columns of Table S1† show that the transitions 1′ → 2 and 1 → 2 with all the molecular complexes are highly exergonic. In the case of Pd_79_, the notable exergonicity of 1 → 2 is primarily due to the substantial exergonicity of PhBr adsorption (1 → 1′). The Δ*G*_1′__→2_ values for the Pd_79_ fac–ed and fac channels are positive (1.9 and 3.2 kcal mol^−1^, respectively), while Δ*G*_1′__→2_ for ed–ed is negative, being −3.8 kcal mol^−1^. However, state 2 in the Pd_79_ fac–ed and ed–ed channels does not correspond to the structures of the OA products observed in the MTD runs (see Fig. 1a). In particular, we can see that most structures of the post-OA complexes at the top of Fig. 1a have Br and Ph chemisorbed on two adjacent (1 0 0) facets.

Such a structure resulting from the facile migration of Br and Ph to the (1 0 0) facets is depicted in Fig. 1a as 3. The values of Δ*G*_1′__→2_ are negative for all three reaction channels. This suggests that the accumulation of chemisorbed Ph and Br (the products of 1′ → 3) can be thermodynamically favorable when higher surface energy (1 0 0) facets are available on the NP surface along with edges between (1 1 1) facets. The availability of (1 0 0) surfaces was previously associated with a higher propensity for Pd leaching and activity in Suzuki cross-coupling.^34^ This final product 3 differs significantly from species 2 in molecular complexes; in the NP system, the Ph and Br groups are separated, whereas in the molecular complexes, they remain bonded to a single Pd center.

By comparing 
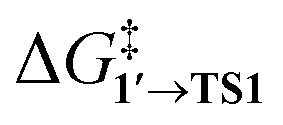
 and Δ*G*_1′__→2_ in Pd_55_, Pd_79_, and Pd_140_ systems, we can assess the effect of the nanoparticle size on the kinetic and thermodynamic feasibility of PhBr oxidative addition. The lowest 
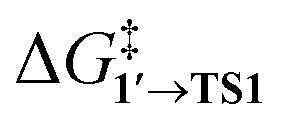
 values for each NP are presented in Table 1, while Table S1† contains all the computed values. The 
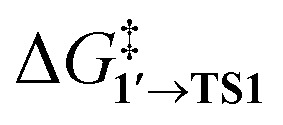
 values indicate that the activity of the edge sites increases with the increase of the nanoparticle size. The non-monotonous behavior of Δ*G*_1′__→2_ with the increase of the nanoparticle's size is also evident, while all Δ*G*_1′__→2_ in Table 1 are negative. Given that the post-OA migration of Ph was observed in all MTD simulations (see Fig. 1, S1 and S2†), and that process 1′ → 3 was highly exergonic in the Pd_79_ system, we may expect the high propensity of Pd NPs to undergo the OA of PhBr when there is a sufficient surface density of edge sites and presence of (1 0 0) surfaces. At the same time, the fraction of edge atoms is smaller in larger nanoparticles; hence, the density of active sites decreases with the increase in the size.

**Table 1:** Computed free (activation) energies of oxidative addition to Pd_55_, Pd_79_, and Pd_140_ for the sites having the lowest 
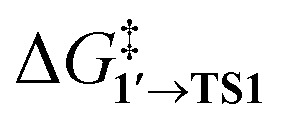
 for each NP

System	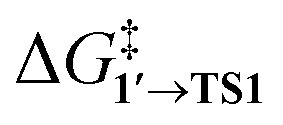	Δ*G*_1′__→2_
Pd_55_ ed	7.5	−6.0
Pd_79_ ed–ed	6.0	−3.8
Pd_140_ ed	3.9	−9.7

Although previous experimental studies associated OA of PhBr with the leaching of Pd into the solution,^11,18,47^ within the context of this study, we can only hypothesize that Ph and Br on the surface may kinetically facilitate this process. Notably, our earlier analysis, which considered only thermodynamic factors, indicated that OA leads to negative (favorable) formation energies of molecular forms (metal complexes) of leached Pd in solution.^17^ Additionally, the spontaneous formation of Pd NPs is a common phenomenon in coupling reactions where the (pre)catalyst is initially introduced as a metal complex. Therefore, OA to Pd NPs should be recognized as a crucial mechanistic step. OA to Pd NPs is as kinetically feasible as the OA to Pd(0) complexes, reinforcing the concept of the catalytic “cocktail”, in which a variety of potentially active and inactive interconverting centers contribute to the overall reaction outcome.

## 3. Discussion and conclusions

**Fig. 4:**
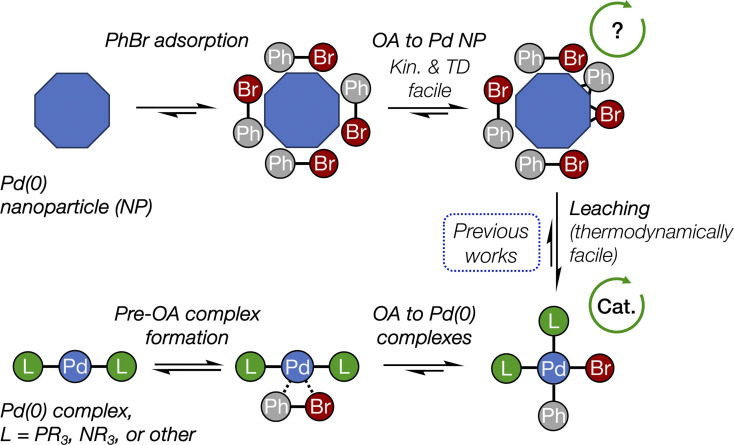
The interplay of oxidative addition in catalyst interconversions in the course of (cross-)couplings with Pd(0) nanoparticles and molecular complexes. See Fig. 2 for more detailed structures and Fig. 3 for reaction (activation) free energies. The arrowed circles represent (possible) participation in the catalytic cycle (see text for discussion).

The conclusions of this study are schematically depicted in Fig. 4, which highlights the interplay of the elucidated transformations in the (cross-)coupling catalysis of reactions involving aryl halides. In our DFT modeling, we analyzed the activity of the nanoparticles ranging from ∼1.08 (Pd_55_) to ∼1.56 nm (Pd_140_). We focused primarily on the edges between their (1 1 1) facets that exhibit the highest thermodynamic stability;^48^ however, we recognize that other centers containing low-valent Pd atoms (*e.g.*, various vertices and kinks) could also be active in OA.

Pd nanoparticles featuring a sufficient surface concentration of low-coordinated atoms, such as facet edges, and atoms on higher-surface energy facets, such as (1 0 0), can undergo oxidative addition of aryl halides. The presented DFT modeling suggests that OA to Pd nanoparticles is well comparable to OA to Pd(0) complexes by its activation barrier and also being thermodynamically favorable. (1 1 1) terraces on Pd nanoparticles also showed relatively high activity, though lower than that of the considered edge sites. OA to all nanoparticles was both kinetically facile at 25 °C and thermodynamically favorable.

For the first time, the present study revealed the critical difference between the OA involving monometallic centers, *i.e.*, molecular complexes, and metal nanoparticles. Classic OA to a monometallic Pd center proceeds as reversible coordination of Ph–Br followed by three-center interaction and breakage of C–Br bond with both Ph and Br groups remaining in close proximity. In contrast, OA to Pd NPs involves practically irreversible trapping of Ph–Br on the metal surface followed by facile C–Br bond breakage and separation of Ph and Br from each other *via* multicentered interactions on the surface.

The debate^49^ about the occurrence of a (cross-)coupling process at the nanoparticle surface continues in recent literature, with works reporting the reaction at the metal surface,^50,51^ exclusively in solution,^33,52^ or in both modes, depending on the reaction conditions.^53^ Here, we show that the OA stage alone would not hamper (cross-)coupling at the nanoparticle surface, according to its calculated reaction (activation) free energies that are comparable with those related to Pd(0) complexes. Another important point is that the type of the ligand, Pd ligation state, type of the organic substrate, and even the solvent can strongly affect the mechanism, kinetics, and selectivity of OA to metal complexes.^23,28,29,54–61^ Therefore, the relative activity of Pd complexes and nanoparticles in OA depends on the choice of reference ligand. The rate of the OA to Pd complexes bearing some designer ligands may surpass that to Pd NPs. In addition, mass transfer effects may be at play in the case of aryl halides OA to Pd NPs. A definitive answer to the question of the feasibility of truly heterogeneous (cross-)coupling on Pd(0) surface requires further investigation, particularly regarding the surface activity of Pd NPs in other coupling steps, such as transmetalation and reductive elimination.

The discussions in the present study highlight many significant phenomena related to coupling reactions involving Pd nanoparticles that require further elucidation. This finding emphasized that our understanding of oxidative addition is still evolving and incomplete.

## 4. Computational details

GFN1-xTB^62^ was utilized for MTD simulations and thermochemical correction calculations in ASE.^63^ The GBSA solvation model parameterized for GFN1-xTB was employed to account for solvent effects.^64^ MTD simulations were conducted using DFTB+ 22.2 (ref. 65) and PLUMED 2.8.2.^66,67^ DFT calculations were carried out using the revPBE functional^68^ with the D3(BJ) correction^69,70^ in VASP 6.3.2.^71^ Core electron density was modeled using PAW.^72^ The single-point Hessian (SPH) method^73^ implemented in the xtb program^64^ was used to perform thermochemical calculations. Additional computational details are provided in the ESI.† ChatGPT 4 was used for initial text proofreading.

## Data availability

The data supporting this article have been uploaded as part of the ESI.†

## Author contributions

Mikhail V. Polynski: conceptualization; formal analysis; investigation; methodology; supervision; writing − original draft; writing − review & editing. Yulia S. Vlasova: formal analysis; investigation; writing − original draft. Yaroslav V. Solovev: formal analysis; investigation; visualization. Sergey M. Kozlov: resources; writing − original draft; writing − review & editing. Valentine P. Ananikov: conceptualization; supervision; writing − original draft, writing − review & editing.

## Conflicts of interest

There are no conflicts of interest to declare.

## Supplementary Material

SC-015-D4SC00628C-s001

SC-015-D4SC00628C-s002

SC-015-D4SC00628C-s003
