# Supplementary material for: Computational analysis of R–X oxidative addition to Pd nanoparticles
Source: Chem Sci. 2024 May 24;15(26):9977–86. doi: 10.1039/d4sc00628c (PMC11220582; doi:10.1039/d4sc00628c)
Supplement: SC-015-D4SC00628C-s001 [file SC-015-D4SC00628C-s001.pdf]

Electronic Supplementary Material (ESI) for Chemical Science.
This journal is © The Royal Society of Chemistry 2024

Supporting Information for the Article

Computational Analysis of R-X Oxidative Addition to Pd Nanoparticles

Mikhail V. Polynski[1], Yulia S. Vlasova[2,3], Yaroslav V. Solovev[4], Sergey M. Kozlov[1], Valentine P. Ananikov*[2,3]*

[1] Department of Chemical and Biomolecular Engineering, National University of Singapore, 4 Engineering Drive 4, Singapore 117585, Singapore.

[2] Faculty of Chemistry, Moscow State University, Leninskiye Gory 1-3, Moscow 119991, Russia.

[3] Zelinsky Institute of Organic Chemistry, Russian Academy of Sciences, Leninsky Prospect 47, Moscow 119991, Russia.

[4] M.M. Shemyakin and Yu.A. Ovchinnikov Institute of Bioorganic Chemistry of the Russian Academy of Sciences, Miklukho-Maklaya 16/10, Moscow 117997, Russia.

AUTHOR INFORMATION

Corresponding Authors

* polynskimikhail@gmail.com, mvp@nus.edu.sg (M.V.P.); val@ioc.ac.ru (V.P.A.).

Table of Contents

1. Computational Details S3
2. The Results of the Metadynamics Simulations of the PhBr Oxidative Addition to Pd Nanoparticles S8
3. Free (Activation) Energies of the PhBr Oxidative Addition to Pd Complexes and Nanoparticles S10
4. References.. S11

1. Computational Details

Born-Oppenheimer molecular dynamics (MD) and metadynamics (MTD) simulations were conducted using the DFTB+ 22.2[1] program. To run well-tempered metadynamics,[2] we employed PLUMED 2.8.2.[3,4] interfaced with DFTB+. In all simulations, a time step of 0.5 fs was selected, and the velocity Verlet driver was used. All MD and MTD trajectories were run for 100 ps. Berendsen thermostat (T = 298.15 K)[5] was used with a time constant equal to 100 fs in MD and MTD simulations. Atomic positions and velocities were recorded every 20 steps. The GFN1-xTB Hamiltonian[6] was used. The convergence criterion for the self-consistent field (SCF) procedure was set to 10^{-6} Ha. During the SCF procedure, the Broyden mixing scheme[7] was used along with the Divide-And-Conquer solver. Fermi filling of electronic states was used with the temperature 298.15 K. GBSA was used to model bulk solvent effects in all MD and MTD simulations (standard parameters for water were used).[8] To construct an initial model system, we put a PhBr molecule on top of either Pd_{55}, Pd_{79}, or Pd_{140} nanoparticle (NP) at a distance enabling contact between van-der-Waals spheres around PhBr and the NP. The initial system was subjected to geometry optimization and subsequent MD to reach thermal equilibrium and sample atomic positions and velocities.

Well-tempered metadynamics parameters were as follows. The distance between the C1 atom of the Ph group and the Br atom was selected as the collective variable. Atomic positions and velocities from MD snapshots with a time gap of no less than 10 ps were used as the initial states in the MTD runs. The bias factor, temperature, and initial hill addition frequency were set to 4, 298.15 K, and 200, respectively. The width of the Gaussian-type bias potential (SIGMA) was 0.01 nm. In the Pd_{79} system, the height of the bias potential (HEIGHT) was varied in decreasing order: 4.184, 4.184/2, 4.184/4, 4.184/6, 4.184/8 kJ/mol, given in the original units of the

program. After examining the output trajectories, it was found that while the value of 4.184/6 kJ/mol allows observing an oxidative addition event in the Pd_{79} system in 5/6 cases after several 10s of ps (Figure 1, main text), the value of 4.184/8 kJ/mol did not lead to an OA event. Therefore, HEIGHT equal to 4.184/6 kJ/mol was selected for final consideration. The same occurrence rate of OA events, 5 out of 6, was observed in the Pd_{55} system. In contrast, only one OA event was observed in the Pd_{140} system within 100 ps of sampling, attributed to the increased size of the configuration space.

To obtain initial geometries for DFT calculations, the XTB ASE[9] calculator was used. The same GFN1-xTB Hamiltonian was used for this purpose. Starting geometries of the final and initial states in these semi-empirical calculations were obtained by taking snapshot structures closest to a transition state in an MTD trajectory and optimizing the geometries after perturbing the near-TS structure in two opposite directions along the reaction coordinate. The electronic temperature of the XTB calculator was set to 300.0 K.

In the calculations with the semi-empirical GFN1-xTB method, the BFGS algorithm with a line search mechanism (BFGSLineSearch) was used with the convergence criterion equal to 0.01 eV/Å for the maximal force component to optimize the initial and final states. *Initial* transition state structures were determined using the scaled dynamic NEB algorithm (DyNEB).[10] 9 images were used in all NEB calculations. After an initial DyNEB run with the convergence criterion equal to 0.1 eV/Å for the maximal force component, the climbing-image procedure was initiated with the following parameters. The spring constant value was 1.0 eV/Å. The FIRE algorithm[11] was used for geometry optimization with the threshold for the geometry step equal to 0.01 Å (maxstep = 0.01) and the convergence criterion of 0.03 eV/Å for the maximal force component. The DyNEB parameters scale_fmax and fmax were set to 2.0 and 0.01 eV/Å, respectively.

All obtained PES minima and saddle points were subjected to vibrational frequency analysis (delta = 0.01 Å, nfree = 2). In all minima, no imaginary modes were found, except for occasional minor (< $10i$ cm^{-1}) artifact modes corresponding to the translation or rotation of the system as a whole. In all transition states, only one imaginary mode corresponding to the vibration along the reaction coordinate was found, except for the occasional minor artificial translational or rotational modes mentioned above.

Spin-polarized DFT calculations with the revPBE functional [12] were performed in the VASP 6.3.2 program.[13] This GGA functional was selected due to the previously demonstrated accuracy of GGA functionals in predicting metal cohesive energies, bond lengths, and bulk moduli, which generally surpass that of hybrid functionals, [14] as well as on the proven accuracy of revPBE in predicting the chemisorption energetics of atoms and molecules on transition-metal surfaces. [15] The D3 dispersion corrections with the Becke-Johnson damping functions were used.[16,17] The energy cutoff for the plane-wave basis set was equal to 415.0 eV. The PAW method [18] was used to model core electron density. The Fermi-Dirac smearing was employed with the smearing width parameter equal to 0.03 eV. SCF and ionic relaxation convergence criteria were set to 10^{-5} eV and 0.01 eV/Å for the maximal force component. The ALGO and PREC parameters were set to "Normal." The symmetry consideration was switched off (ISYM = −1). The FIRE algorithm implemented in the VTST tools was employed. Non-spherical contributions related to electron density gradient in PAW spheres were included (LASPH = .TRUE.). All systems were treated as orthorhombic with cell vectors equal to 22.1, 26.1, and 21.1 Å (so that the largest system among those considered was separated from its periodic images by no less than 10.0 Å).

The final structures of transition states were optimized at the DFT level using the DIMER method implemented in the VTST tools.[19–22] During this procedure, structures initially optimized

using GFN1-xTB were taken as starting points, and the vectors of the imaginary mode from the semi-empirical calculations were used to set the initial direction along the dimer. After each TS optimization, we perturbed the structure of the TS in opposite directions along the reaction coordinate and then subjected the perturbed structures to geometry optimization to re-evaluate (and correct, if necessary) the structures of the initial and final states. The ionic relaxation convergence criterion was set to 0.03 eV/Å for the maximal force component. All other parameters in the DIMER calculations were set to the same values as stated above. All found saddle points were subjected to vibrational frequency analysis. No significant imaginary modes were found except those representing the vibrations along the reaction coordinate (TS modes). Among occasional insignificant imaginary modes, only those related to translational or rotational motion of the system as a whole or those related to slight under-relaxation of ionic positions (< 20 cm^{-1}) were found. As long as a reasonably tight convergence criterion was used during the geometry optimizations, these spurious modes were disregarded.

Thermochemical calculations were conducted employing the GFN1-xTB Hamiltonian and the single-point Hessian (SPH) method[23] implemented in the xtb program.[8] For each species A, its Gibbs free energy was calculated according to the equation:

$$G_A^{vib} = E_A^{el,DFT} + \Delta G_A^{SPH} + \Delta G_A^{solv} + \Delta G^{IG-sol},$$

Where $E_A^{el,DFT}$ is the full electronic energy of system A, including dispersion corrections, computed with the selected DFT methodology; ΔG_A^{SPH} includes all enthalpic and entropic SPH corrections—rotational, translational, and harmonic vibrational—at the selected temperature (298.15 K); ΔG_A^{solv} is the free energy of solvation in water computed using the GBSA model[8] as described below; ΔG^{IG-sol} is the term equal to 1.89 kcal/mol, corresponding to the change in free energy upon the transition of the system from ideal gas to 1M solution. It should be noted that

low-frequency vibrational modes computed within the SPH approach are treated as quasi-rotations within a widely used model for vibrational entropy, the use of which is associated with increased accuracy in thermochemical calculations.[24] The ΔG_A^{solv} term was computed as follows:

$$\Delta G_A^{solv} = E_A^{GFN1-GBSA} - E_A^{GFN1},$$

Where $E_A^{GFN1-GBSA}$ is the single-point energy of A computed with GFN1-xTB, and the GBSA model applied; E_A^{GFN1} is the energy of A optimized in vacuum using GFN1-xTB.

2. The Results of the Metadynamics Simulations of the PhBr Oxidative Addition to Pd Nanoparticles

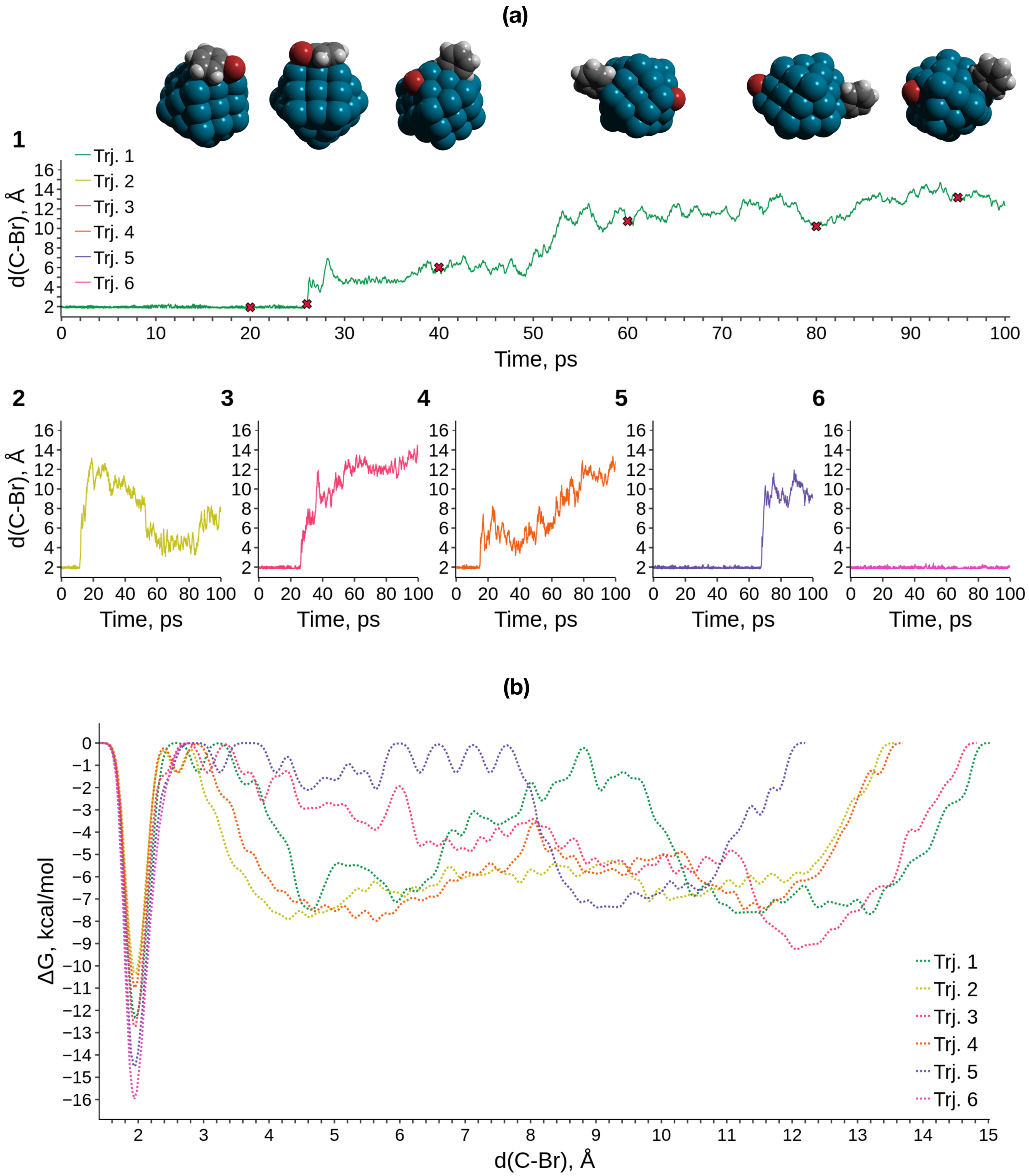

Figure S1. Collective variable (C-Br distance) evolution during the metadynamics simulations of the Pd_{55} system; (b) Free energy profiles obtained from the metadynamics runs.

(a)

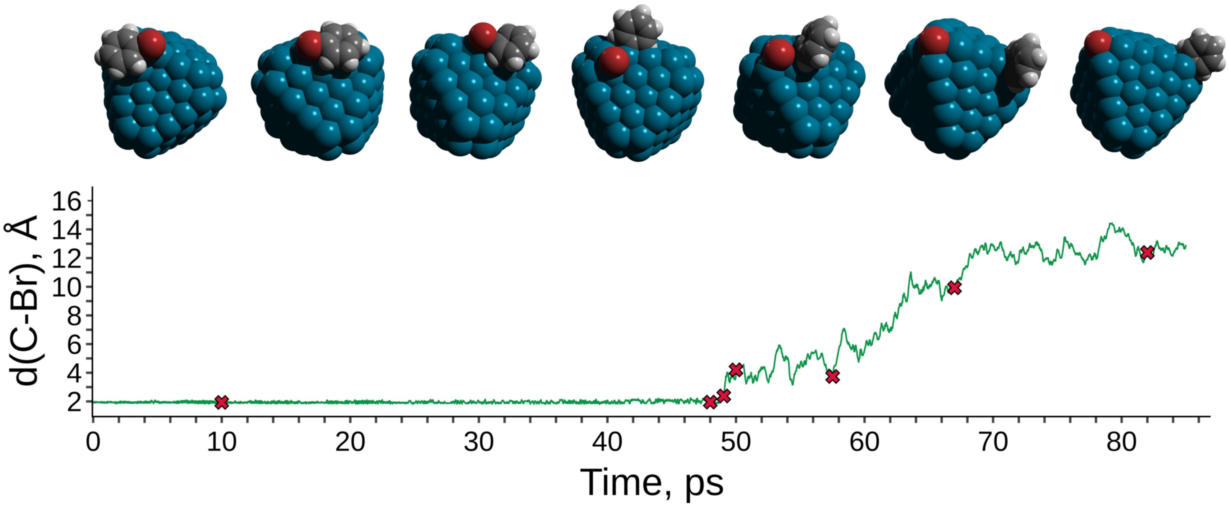

(b)

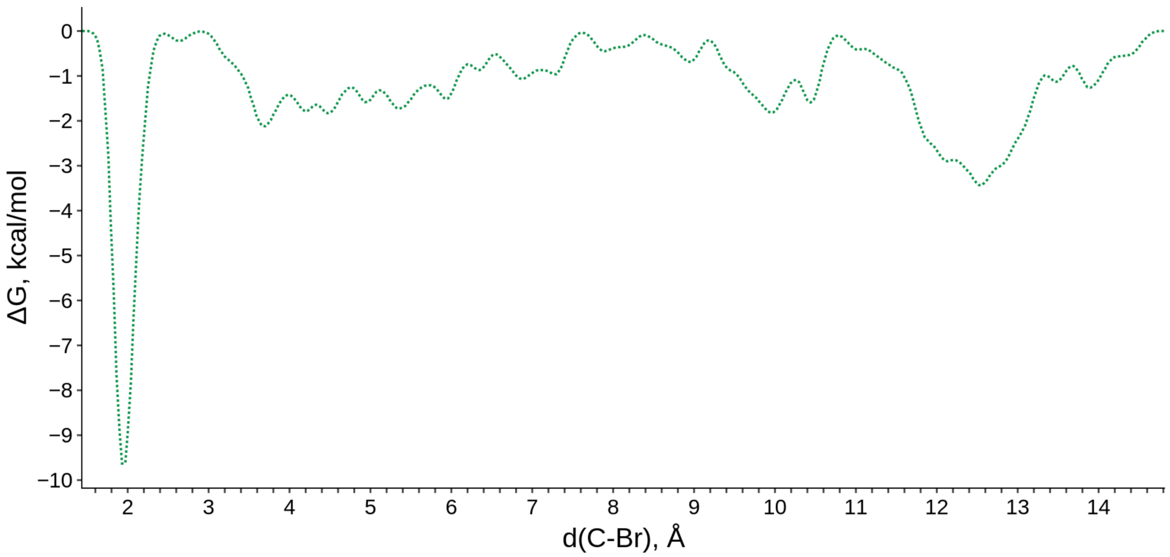

Figure S2. Collective variable (C-Br distance) evolution during the metadynamics simulation of the Pd_{140} system; (b) Free energy profile obtained from the metadynamics run.

3. **Free (Activation) Energies of the PhBr Oxidative Addition to Pd Complexes and Nanoparticles**

Table S1. Model mechanism of oxidative addition and free (activation) energies of the corresponding elementary steps in kcal/mol.

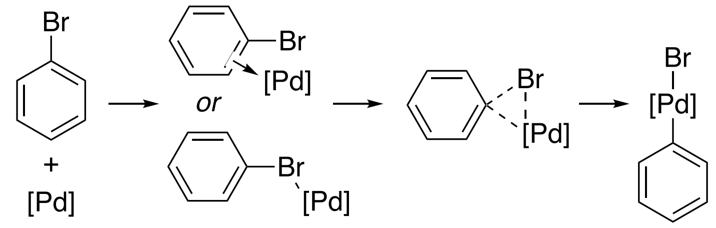

1 **1'** **TS1** **2**

System	$\Delta G_{1\to1'}$	$\Delta G^{\ddagger}_{1'\to TS1}$	$\Delta G_{1'\to2}$	$\Delta G_{1\to2}$	$\Delta G_{2\to3}$	$\Delta G_{1'\to3}$
Pd_{55} ed		7.5	−6.0			
Pd_{55} ver		9.2	−18.0			
Pd_{79} ed-ed	−49.7	6.0	−3.8	−53.5	−7.1	−10.8
Pd_{79} fac-ed	−48.1	11.4	1.9	−46.2	−14.3	−12.4
Pd_{79} fac	−46.9	13.4	3.2	−43.7	−16.8	−13.6
Pd_{140} ed		3.9	−9.7			
L = NEt_3	−12.0	9.7	−20.8	−32.9		
L = PMe_3	5.0	7.6	−32.1	−27.0		
L = PPh_3	−1.9	11.5	−25.4	−27.2		

4. References

(1) Hourahine, B.; Aradi, B.; Blum, V.; Bonafé, F.; Buccheri, A.; Camacho, C.; Cevallos, C.; Deshaye, M. Y.; Dumitric, T.; Dominguez, A.; Ehlert, S.; Elstner, M.; Van Der Heide, T.; Hermann, J.; Irle, S.; Kranz, J. J.; Köhler, C.; Kowalczyk, T.; Kubař, T.; Lee, I. S.; Lutsker, V.; Maurer, R. J.; Min, S. K.; Mitchell, I.; Negre, C.; Niehaus, T. A.; Niklasson, A. M. N.; Page, A. J.; Pecchia, A.; Penazzi, G.; Persson, M. P.; Å&tild;ezáč, J.; Sánchez, C. G.; Sternberg, M.; Stöhr, M.; Stuckenberg, F.; Tkatchenko, A.; Yu, V. W. Z.; Frauenheim, T. DFTB+, a Software Package for Efficient Approximate Density Functional Theory Based Atomistic Simulations. *Journal of Chemical Physics* **2020**, *152* (12), 124101. https://doi.org/10.1063/1.5143190/953756.

(2) Barducci, A.; Bussi, G.; Parrinello, M. Well-Tempered Metadynamics: A Smoothly Converging and Tunable Free-Energy Method. *Phys Rev Lett* **2008**, *100* (2), 020603. https://doi.org/10.1103/PHYSREVLETT.100.020603/FIGURES/3/MEDIUM.

(3) Tribello, G. A.; Bonomi, M.; Branduardi, D.; Camilloni, C.; Bussi, G. PLUMED 2: New Feathers for an Old Bird. *Comput Phys Commun* **2014**, *185* (2), 604–613. https://doi.org/10.1016/J.CPC.2013.09.018.

(4) Bonomi, M.; Bussi, G.; Camilloni, C.; Tribello, G. A.; Banáš, P.; Barducci, A.; Bernetti, M.; Bolhuis, P. G.; Bottaro, S.; Branduardi, D.; Capelli, R.; Carloni, P.; Ceriotti, M.; Cesari, A.; Chen, H.; Chen, W.; Colizzi, F.; De, S.; De La Pierre, M.; Donadio, D.; Drobot, V.; Ensing, B.; Ferguson, A. L.; Filizola, M.; Fraser, J. S.; Fu, H.; Gasparotto, P.; Gervasio, F. L.; Giberti, F.; Gil-Ley, A.; Giorgino, T.; Heller, G. T.; Hocky, G. M.; Iannuzzi, M.; Invernizzi, M.; Jelfs, K. E.; Jussupow, A.; Kirilin, E.; Laio, A.; Limongelli, V.; Lindorff-Larsen, K.; Löhr, T.; Marinelli, F.; Martin-Samos, L.; Masetti, M.; Meyer, R.; Michaelides, A.; Molteni, C.; Morishita, T.; Nava, M.; Paissoni, C.; Papaleo, E.; Parrinello, M.; Pfaendtner, J.; Piaggi, P.; Piccini, G. M.; Pietropaolo, A.; Pietrucci, F.; Pipolo, S.; Provasi, D.; Quigley, D.; Raiteri, P.; Raniolo, S.; Rydzewski, J.; Salvalaglio, M.; Sosso, G. C.; Spiwok, V.; Šponer, J.; Swenson, D. W. H.; Tiwary, P.; Valsson, O.; Vendruscolo, M.; Voth, G. A.; White, A. Promoting Transparency and Reproducibility in Enhanced Molecular Simulations. *Nature Methods 2019 16:8* **2019**, *16* (8), 670–673. https://doi.org/10.1038/s41592-019-0506-8.

(5) Berendsen, H. J. C.; Postma, J. P. M.; Van Gunsteren, W. F.; Dinola, A.; Haak, J. R. Molecular Dynamics with Coupling to an External Bath. *J Chem Phys* **1984**, *81* (8), 3684–3690. https://doi.org/10.1063/1.448118.

(6) Grimme, S.; Bannwarth, C.; Shushkov, P. A Robust and Accurate Tight-Binding Quantum Chemical Method for Structures, Vibrational Frequencies, and Noncovalent Interactions of Large Molecular Systems Parametrized for All Spd-Block Elements (Z = 1–86). *J Chem Theory Comput* **2017**, *13* (5), 1989–2009. https://doi.org/10.1021/acs.jctc.7b00118.

(7) Johnson, D. D. Modified Broydens Method for Accelerating Convergence in Self-Consistent Calculations. *Phys Rev B* **1988**, *38* (18), 12807–12813. https://doi.org/10.1103/PhysRevB.38.12807.

(8) Bannwarth, C.; Caldeweyher, E.; Ehlert, S.; Hansen, A.; Pracht, P.; Seibert, J.; Spicher, S.; Grimme, S. Extended Tight-Binding Quantum Chemistry Methods. *Wiley Interdiscip Rev Comput Mol Sci* **2021**, *11* (2), e1493. https://doi.org/10.1002/WCMS.1493.

(9) Hjorth Larsen, A.; JØrgen Mortensen, J.; Blomqvist, J.; Castelli, I. E.; Christensen, R.; Dułak, M.; Friis, J.; Groves, M. N.; Hammer, B.; Hargus, C.; Hermes, E. D.; Jennings, P.

C.; Bjerre Jensen, P.; Kermode, J.; Kitchin, J. R.; Leonhard Kolsbjerg, E.; Kubal, J.; Kaasbjerg, K.; Lysgaard, S.; Bergmann Maronsson, J.; Maxson, T.; Olsen, T.; Pastewka, L.; Peterson, A.; Rostgaard, C.; SchiØtz, J.; Schütt, O.; Strange, M.; Thygesen, K. S.; Vegge, T.; Vilhelmsen, L.; Walter, M.; Zeng, Z.; Jacobsen, K. W. The Atomic Simulation Environment—a Python Library for Working with Atoms. *Journal of Physics: Condensed Matter* **2017**, *29* (27), 273002. https://doi.org/10.1088/1361-648X/AA680E.
(10) Lindgren, P.; Kastlunger, G.; Peterson, A. A. Scaled and Dynamic Optimizations of Nudged Elastic Bands. *J Chem Theory Comput* **2019**, *15* (11), 5787–5793. https://doi.org/10.1021/ACS.JCTC.9B00633/SUPPL_FILE/CT9B00633_SI_002.MP4.
(11) Bitzek, E.; Koskinen, P.; Gähler, F.; Moseler, M.; Gumbsch, P. Structural Relaxation Made Simple. *Phys Rev Lett* **2006**, *97* (17), 170201. https://doi.org/10.1103/PHYSREVLETT.97.170201/FIGURES/2/MEDIUM.
(12) Zhang, Y.; Yang, W. Comment on "Generalized Gradient Approximation Made Simple." *Phys Rev Lett* **1998**, *80* (4), 890. https://doi.org/10.1103/PhysRevLett.80.890.
(13) Kresse, G.; Furthmüller, J. Efficient Iterative Schemes for *Ab Initio* Total-Energy Calculations Using a Plane-Wave Basis Set. *Phys Rev B* **1996**, *54* (16), 11169–11186. https://doi.org/10.1103/PhysRevB.54.11169.
(14) Janthon, P.; Luo, S.; Kozlov, S. M.; Viñes, F.; Limtrakul, J.; Truhlar, D. G.; Illas, F. Bulk Properties of Transition Metals: A Challenge for the Design of Universal Density Functionals. *J Chem Theory Comput* **2014**, *10* (9), 3832–3839. https://doi.org/10.1021/ct500532v.
(15) Hammer, B.; Hansen, L. B.; Nørskov, J. K. Improved Adsorption Energetics within Density-Functional Theory Using Revised Perdew-Burke-Ernzerhof Functionals. *Phys Rev B* **1999**, *59* (11), 7413. https://doi.org/10.1103/PhysRevB.59.7413.
(16) Grimme, S.; Antony, J.; Ehrlich, S.; Krieg, H. A Consistent and Accurate *Ab Initio* Parametrization of Density Functional Dispersion Correction (DFT-D) for the 94 Elements H-Pu. *J Chem Phys* **2010**, *132* (15), 154104. https://doi.org/10.1063/1.3382344.
(17) Grimme, S.; Ehrlich, S.; Goerigk, L. Effect of the Damping Function in Dispersion Corrected Density Functional Theory. *J Comput Chem* **2011**, *32* (7), 1456–1465. https://doi.org/10.1002/jcc.21759.
(18) Kresse, G.; Joubert, D. From Ultrasoft Pseudopotentials to the Projector Augmented-Wave Method. *Phys Rev B* **1999**, *59* (3), 1758–1775. https://doi.org/10.1103/PhysRevB.59.1758.
(19) Henkelman, G.; Jónsson, H. A Dimer Method for Finding Saddle Points on High Dimensional Potential Surfaces Using Only First Derivatives. *J Chem Phys* **1999**, *111* (15), 7010–7022. https://doi.org/10.1063/1.480097.
(20) Heyden, A.; Bell, A. T.; Keil, F. J. Efficient Methods for Finding Transition States in Chemical Reactions: Comparison of Improved Dimer Method and Partitioned Rational Function Optimization Method. *Journal of Chemical Physics* **2005**, *123* (22). https://doi.org/10.1063/1.2104507/776452.
(21) Kästner, J.; Sherwood, P. Superlinearly Converging Dimer Method for Transition State Search. *Journal of Chemical Physics* **2008**, *128* (1). https://doi.org/10.1063/1.2815812/928095.
(22) Xiao, P.; Sheppard, D.; Rogal, J.; Henkelman, G. Solid-State Dimer Method for Calculating Solid-Solid Phase Transitions. *Journal of Chemical Physics* **2014**, *140* (17). https://doi.org/10.1063/1.4873437/317097.

(23) Spicher, S.; Grimme, S. Single-Point Hessian Calculations for Improved Vibrational Frequencies and Rigid-Rotor-Harmonic-Oscillator Thermodynamics. *J Chem Theory Comput* **2021**, *17* (3), 1701–1714. https://doi.org/10.1021/ACS.JCTC.0C01306/SUPPL_FILE/CT0C01306_SI_002.ZIP.
(24) Grimme, S. Supramolecular Binding Thermodynamics by Dispersion-Corrected Density Functional Theory. *Chemistry - A European Journal* **2012**, *18* (32), 9955–9964. https://doi.org/10.1002/chem.201200497.
